# Editorial: Metabolism in the tumour microenvironment: implications for pathogenesis and therapeutics

**DOI:** 10.3389/fimmu.2026.1788008

**Published:** 2026-01-27

**Authors:** Adil Rasheed, Fabrizio Fontana

**Affiliations:** 1 Immunology Center of Georgia, Medical College of Georgia, Augusta University, Augusta, GA, United States; 2 Department of Physiology, Medical College of Georgia, Augusta University, Augusta, GA, United States; 3 Department of Pharmacological and Biomolecular Sciences “Rodolfo Paoletti”, University of Milan, Milan, Italy

**Keywords:** cancer immunotherapy, glutamine, immunoediting, immunometabolism, lactate, metabolism, tumor, tumor microenvironment

The tumour microenvironment (TME) is a heterogeneous composition of stromal, vascular and immune cells that respond to local and systemic cues and influence tumour progression and metastasis (1). Preclinical and clinical evidence demonstrate a strong reciprocal relationship between tumour cell oncogenesis and its TME (2). Rather than serving as a passive backdrop, the TME functions as a metabolic ecosystem that shapes immunosurveillance, immune evasion and responsiveness to therapy (3). Importantly, the articles featured in this Research Topic converge on a shared principle that metabolic rewiring of the TME is an active driver of immune dysfunction which drives tumour growth and metastasis and contributes to the global health burden of cancer.

We curated 11 articles that highlight this rapidly advancing frontier, focused on how metabolism reshapes the TME. Collectively, these contributions synthesize mechanistic advances, discuss translational hurdles and outline opportunities for therapeutic advancement that leverage these findings into clinical practice.

Complementary articles by Wik et al. and Li et al. discuss how metabolic reprogramming during tumour development alters the TME and its characteristics, including nutrient availability, hypoxia and acidity, which together reprogram immune phenotypes to support tumour growth. A central observation is the Warburg effect in tumour cells, which reduces TME glucose availability, blunts effector T-cell responses and impairs anti-tumour immunity. These reviews also describe metabolic dysfunction across other immune cells that support immunosuppressive and pro-tumorigenic phenotypes, including natural killer (NK) cells, macrophages and neutrophils, alongside metabolic cues that stimulate vascularization and metastasis. Li et al. further highlight dynamic tumour–immune cell play, whereby “immunoediting” constitutes selection pressure that favours metabolic phenotypes conducive to immune escape. Beyond solid tumours, this Research Topic features a review by Hao et al. describing metabolic-induced dysfunction in the bone marrow microenvironment and its interaction with leukemic clones during acute myeloid leukaemia (AML). While the bone marrow microenvironment shares features of bi-directional dysfunction with the solid TME, the authors emphasize unique aspects that give rise to leukemic clone selection and expansion. They further discuss AML metabolic dependencies intersecting with differentiation programs and immune suppression, suggesting broader relevance across malignancies and the potential for metabolic interventions as adjunct therapy to improve current immunotherapies, including CAR-T therapy and hematopoietic stem cell transplantation.

A second set of reviews focuses on specific classes of metabolites, including lactate and amino acids, as drivers of TME dysfunction. Together, these articles provide historical and conceptual context for cancer immunotherapy, discuss roadblocks to translate metabolism-focused therapies into the clinic, and outline strategies to overcome these hurdles. A recurring theme is a paradox in which pathways sustaining tumour proliferation and redox homeostasis simultaneously expose therapeutic vulnerabilities of the TME. Metabolites may be produced via the Reverse Warburg Effect, whereby tumour cells promote stromal cells, such as cancer-associated fibroblasts (CAFs), to undergo aerobic glycolysis and release lactate. As discussed by Gu et al. and Yang et al., lactate is not merely a substrate; it acidifies the TME and drives immunosuppressive phenotypes of T cells and NK cells. Additionally, lactate is a substrate for protein modification, including histone lactylation, which influences transcriptional programs, macrophage polarization, and effector T-cell activity, thereby providing a molecular bridge between the Warburg effect and immune tolerance.

Fang et al. add further nuance by discussing metabolic competition between tumour and immune cells, as exemplified by glutamine. In many cancers, tumour cells exhibit “glutamine addiction” that supports survival while depleting glutamine availability to immune cells, such as effector T cells, which impairs anti-tumour functions. Importantly, the authors emphasize context dependence where glutamine exerts differential effects across tumour types and TME immunity. Extending this theme, Jiang et al. broaden the discussion beyond glutamine and examine the importance of amino acid metabolism in gastric cancer and additionally highlight arginine and tryptophan pathways that can enhance antitumor immunity, reverse T-cell exhaustion and synergize with immune checkpoint blockade.

Original research articles in our Research Topic illustrate how metabolic vulnerabilities can be exploited to reshape tumours. Li et al. describe the utility of a small synthetic peptide, RPL41, which reduces retinoblastoma in mice by promoting transcription factor stability and disrupting lysosomal pathways that support growth and metastasis. As a candidate therapeutic, RPL41 small size (25 amino acids) and membrane permeability make it attractive for *in vivo* delivery. Additional studies examine fatty acid and cholesterol metabolism in cancer. Wu et al., leveraging The Cancer Genome Atlas (TCGA) and National Center for Biotechnology Information (NCBI) databases, cell culture and clinical samples, identify fatty acid metabolism–related genes associated with poor prognosis in lung adenocarcinoma. These findings underscore the value of metabolic signatures that may guide risk stratification and predict immunotherapy response. Using metabolomics, Bao et al. profiled serum from lymphoepithelioma-like carcinoma patients and identified elevated linoleic acid levels relative to control. Mechanistic studies in mice linked this to upregulation of tissue factor via peroxisome proliferator–activated receptor α (PPARα), which promotes tumour growth through TME remodelling, including increased immunosuppressive macrophages and suppression of NK cells. Notably, these pro-tumorigenic effects were reversed by pharmacologic tissue factor inhibition, highlighting the potential of modulating metabolic signalling to improve anti-tumour immunity in this lung cancer. Finally, Pan et al., using Genotype–Tissue Expression (GTEx) and TCGA datasets, identify squalene epoxidase (SQLE), an enzyme involved in cholesterol biosynthesis, as highly expressed in pancreatic cancer. SQLE-mediated conversion of squalene promotes tumour escape via infiltration of suppressive myeloid populations and reductions in CD8+ T cells, reinforcing an immunosuppressive TME. These data implicate squalene metabolism as a modifiable axis with potential to reshape the TME and enhance antitumour immunity.

Together, these articles advance several principles:

- Metabolites function as bi-directional communicators tumour cells and their TME
- Targeting TME metabolism may restore or potentiate antitumor immune responses
- Prognostic tools incorporating metabolic features hold strong clinical promise
- Tumour-type-specific metabolic programs will be essential for therapy design

Overall, a cohesive narrative emerges in which tumour and stromal cells undergo metabolic reprogramming that rewires immune effector functions. The field now faces challenges, including metabolic plasticity, systemic host effects and patient heterogeneity, yet opportunities are substantial. Combinations of metabolic inhibitors, immunotherapies, epigenetic modulators and dietary or metabolic interventions with other cancer treatments are likely to underscore the next wave of translational research aimed at durable anti-tumour immunity. Collectively, this Research Topic highlights tumour-specific and dynamically regulated metabolic dependencies that impose dysfunction on the TME and reinforces the rationale for combining metabolic interventions with immunotherapy. Continued integration of single-cell technologies, spatial metabolomics, and systems modelling of clinical samples will be crucial for mapping metabolic crosstalk, identifying patient-specific vulnerabilities, and guiding precision interventions that maximize efficacy while minimizing toxicity across diverse cancer settings across adult and paediatric cancers.
